# Exploring the bi-directional relationship and shared genes between depression and stroke *via* NHANES and bioinformatic analysis

**DOI:** 10.3389/fgene.2023.1004457

**Published:** 2023-03-31

**Authors:** Zhanghuan Yang, Maokun He, Qian Zhang, Shifu Li, Hua Chen, Di Liao

**Affiliations:** ^1^ Department of Oncology, Xiangya Cancer Center, Xiangya Hospital, Central South University, Changsha, Hunan, China; ^2^ Hainan Medical University, Haikou, China; ^3^ Department of Neurosurgery, Xiangya Hospital, Central South University, Changsha, Hunan, China; ^4^ National Clinical Research Center for Geriatric Disorders, Central South University, Changsha, Hunan, China; ^5^ Department of Neurosurgery, The First people’s Hospital of Changde, Changde, China; ^6^ Department of Neurology, Xiangya Hospital, Central South University, Changsha, Hunan, China

**Keywords:** ischemic stroke, major depressive disorder, bioinformatics, shared genes, immune infiltration, NHANES

## Abstract

**Background:** Stroke and depression are the two most common causes of disability worldwide. Growing evidence suggests a bi-directional relationship between stroke and depression, whereas the molecular mechanisms underlying stroke and depression are not well understood. The objectives of this study were to identify hub genes and biological pathways related to the pathogenesis of ischemic stroke (IS) and major depressive disorder (MDD) and to evaluate the infiltration of immune cells in both disorders.

**Methods:** Participants from the United States National Health and Nutritional Examination Survey (NHANES) 2005–2018 were included to evaluate the association between stroke and MDD. Two differentially expressed genes (DEGs) sets extracted from GSE98793 and GSE16561 datasets were intersected to generate common DEGs, which were further screened out in cytoHubba to identify hub genes. GO, KEGG, Metascape, GeneMANIA, NetworkAnalyst, and DGIdb were used for functional enrichment, pathway analysis, regulatory network analysis, and candidate drugs analysis. ssGSEA algorithm was used to analyze the immune infiltration.

**Results:** Among the 29706 participants from NHANES 2005–2018, stroke was significantly associated with MDD (OR = 2.79,95% CI:2.26–3.43, *p* < 0.0001). A total of 41 common upregulated genes and eight common downregulated genes were finally identified between IS and MDD. Enrichment analysis revealed that the shared genes were mainly involved in immune response and immune-related pathways. A protein-protein interaction (PPI) was constructed, from which ten (CD163, AEG1, IRAK3, S100A12, HP, PGLYRP1, CEACAM8, MPO, LCN2, and DEFA4) were screened. In addition, gene-miRNAs, transcription factor-gene interactions, and protein-drug interactions coregulatory networks with hub genes were also identified. Finally, we observed that the innate immunity was activated while acquired immunity was suppressed in both disorders.

**Conclusion:** We successfully identified the ten hub shared genes linking the IS and MDD and constructed the regulatory networks for them that could serve as novel targeted therapy for the comorbidities.

## Introduction

Stroke is the leading cause of death and disability, leading to significant economic losses as a result of its functional impairments (Meschia et al., 2014). Every year more than 795,000 people in the United States (United States) have a stroke, of which 610,000 are first-time strokes, whereas 185,000 patients have previously had a stroke. The vast majority of stroke cases occur as a result of two specific types of stroke (hemorrhagic and ischemic strokes). United States strokes are dominated by ischemic strokes, which account for 87% of all strokes (Barthels and Das, 2020). The prevalence of depression is growing among the general population, typically characterized by anhedonia and the inability to experience pleasure. A variety of somatic symptoms (psychological disturbance, fatigue, and weight fluctuations) as well as cognitive symptoms (poor concentration and negative cognitions) can accompany depression (Bucciarelli et al., 2020). Depression is sub-categorized into major depressive disorder (MDD) and dysthymia. Epidemiologic data indicates that the average lifetime and 12-month prevalence estimates of MDD are 14.6% and 5.5% in high-income and 11.1% and 5.9% in the low-to middle-income countries (Bromet et al., 2011).

Stroke patients are more likely to suffer from depression than the general population. Growing evidence suggests a bi-directional relationship between stroke and depression: 1) depression is associated with an increased risk of stroke (Pan et al., 2011; Wassertheil-Smoller et al., 2018; Harshfield et al., 2020), and 2) depression is particularly prevalent among stroke survivors (Robinson and Jorge, 2016; Das and Rajanikant, 2018). The prevalence of post-stroke depression (PSD) is estimated to be 29% at any time point up to 5 years following a stroke (Hackett and Pickles, 2014). However, the mechanisms underlying the association between depression and stroke are poorly investigated. Biological factors such as alterations in ascending monoamine systems, neuroplasticity, and glutamate neurotransmission and an increasing of pro-inflammatory cytokines were proposed to explain the mechanisms of PSD (Robinson and Jorge, 2016). Moreover, multiple mechanisms may play roles in depression contributing to stroke. First, Smoking, obesity (Ho et al., 2008), and poor health behaviors (i.e., poor diet, physical inactivity, and smoking) (Strine et al., 2008) may increase stroke risk in depression patients. Second, other major comorbidities, such as diabetes (Wesołowska et al., 2018), atherosclerosis (Joynt et al., 2003), and hypertension (Patten et al., 2009), accompanied by depression, are major risk factors for stroke. Finally, the use of antidepressant medication may potentially contribute to the occurrence of stroke events.

Apart from the above-mentioned mechanisms, genetic factors are likely involved in the pathogenesis of depression and stroke. Increased risk for depression in first-degree relatives of depression probands was observed with an estimated odds ratio of 2.84 from a meta-analysis of the highest-quality family studies (Sullivan et al., 2000). The heritability of MDD has been found to be greater in women (42%) than in men (29%) in a Swedish national twin study (Kendler et al., 2006). There are multiple risk factor genes that were thought to participate in the pathogenesis of depression with extremely complex, polygenic, and epistatic inheritance patterns (Zhao et al., 2019a). There is significant evidence that stroke has a hereditary component based on studies of twins, siblings, and families (Humphries and Morgan, 2004). Heritability for all IS is estimated to be 37.9% (Bevan et al., 2012). The heritability of stroke subtypes varies markedly, with 40.3% for large vessels and 32.6% for cardioembolics but lower for cardioembolic small vessels (16.1%). The genetic involvement in the pathogenesis of both stroke and depression as well as the comorbidity frequency is not yet fully established or whether common overlapping genes and biological mechanisms are subserving both disorders.

A common transcription feature may provide new insights into the pathogenesis of depression and stroke. This study aims to identify hub genes and biological pathways related to the pathogenesis of IS and MDD. Furthermore, as increasing evidence points to the involvement of an immune response in both disorders (Beurel et al., 2020; Iadecola et al., 2020), we evaluate the immune cell infiltration and identify the common immune cells.

## Materials and methods

### Dataset collection and processing

The data used in the present work was downloaded from the National Health and Nutrition Examination Survey (NHANES) (https://www.cdc.gov/nchs/index.htm) and the Gene Expression Omnibus (GEO) database (https://www.ncbi.nlm.nih.gov/geo/) based on a microarray or RNA-seq dataset of major depressive disorder (MDD) and ischemic stroke (IS). The NHANES is a research project aimed to assess the health and nutritional status of adults and children in the United States, combining interviews and physical examinations to provide vital and health statistics. The GSE98793 microarray profile included 128 MDD whole blood samples and 64 health samples at the platform of GPL570 Affymetrix U133_Plus2.0 Genechips. The effect of two batches in the GSE98793 dataset were removed by applying removed BatchEffect function of the limma package (Ritchie et al., 2015). The GSE76826 dataset is a microarray profile at the platform of GPL17077 Agilent-039494 SurePrint G3 Human GE v2 8 × 60 K Microarray 039381. The GSE16561 microarray profile contained whole blood from 39 IS patients and was compared with 24 healthy control subjects, measured using a GPL6883 Illumina HumanRef-8 v3.0 expression beadchip. The GSE122709 dataset (including 10 peripheral blood mononuclear cells samples of IS patients and five controls) is a RNA-sequencing dataset and measured at GPL20795 HiSeq X Ten. When multiple probes were matched with one gene, the probe with the highest expression values was annotated in the homologous gene symbol based on the annotation information on the platform.

### NHANES

Data of 70190 participants were available in NHANES 2005–2018. Age, sex, race or ethnicity, education level, poverty, marital status, smoking status, stroke, body mass index (BMI), waist circumference and diabetes was included as variables in the analysis. Depression was measured using the Patient Health Questionnaire (PHQ-9). Participants with PHQ-9 total scores≥10 were considered as having MDD. After excluding participants with missing data, 29706 participants were included in our analysis. Continuous variables are presented as the mean (standard deviation), and categorical variables are presented as the frequency (percentage). The chi-square test or Student’s *t*-test were performed to evaluate the differences between the non-exposure and exposure condition on stroke and MDD. Logistic regression models were performed to calculate odds ratios (ORs) for stroke and MDD.

### Identification of differentially expressed genes (DEGs)

After the data standardization and normalization of datasets using the normalizeBetweenArrays function in the “limma” R package, a principal component analysis (PCA) was conducted by using the “factoextra” R package. The DEGs between cases and healthy controls were analyzed by using the “limma” R package. The criteria of *p*-value <0.05 and |log fold change (FC)|> 0.2 were used to screen the DEGs of MDD and controls, and |log FC| > 0.5 were regarded as cut-off criteria for significant DEGs for IS patients and controls. A volcano plot and a heat map plot were performed by using the R software ggplot2 package (Ginestet, 2011) and “ComplexHeatmap” (Gu et al., 2016) to show significant DEGs, respectively.

### Screening of communal DEGs of MDD and IS

After having separately identified the DEGs of MDD and IS, we intersected their DEGs to screen out the communal DEGs that may participate in the pathogenesis of the two diseases. Only when the DEGs had the same expression trends in both diseases were these common genes kept. The processes were conducted and visualized using the “ggVennDiagram” package (Gao et al., 2021). The overlapped genes were further shown in two disorders with a heat map from the perspective of logFC and *p*-value.

### Function enrichment analysis

The “clusterProfiler” package (Yu et al., 2012) was used to enrich the biological processes (BP), cellular components (CC), and molecular function (MF) of Gene Ontology (GO) (Gene Ontology Consortium, 2015) and Kyoto Encyclopedia of Genes and Genomes (KEGG) pathways (Kanehisa and Goto, 2000) of common DEGs.

### Protein-protein interaction (PPI) network

To detect potential relationships among the DEG-encoded proteins common to both MDD and IS, a protein-protein interaction (PPI) network was constructed using the Search Tool for the Retrieval of Interacting Genes database (STRING, www.string-db.org) (Szklarczyk et al., 2019). Low confidence of 0.15 was set to find more interactions between proteins. The other parameters were set to the default values (i.e., a full STRING network for nerwork type; evidence for meaning of network edges; and all active interaction sources). The contructed network was imported into Cytoscape to be visualized and further analyzed.

### Selection and analysis of hub genes

In this work, we used six common algorithms MCC (Maximal Clique Centrality), MNC (Maximum neighborhood component), DMNC (Density of Maximum Neighborhood Component), Degree, Closeness, and Betweenness) in the cytoHubba plugin of Cytoscape to evaluate and identify hub genes. The detailed information about the six algorithms were descripted in previous article (Chin et al., 2014). The relationships among genes were calculated using Pearson’s correlation methods. The GSE76826 and GSE122709 datasets was applied to validate the expression levels of hub genes.

Subsequently, a co-expression network of these hub genes was constructed *via* GeneMANIA (http://www.genemania.org/) (Warde-Farley et al., 2010), and their potential functional processes were enriched using the Metascape tool (https://metascape.org/) (Zhou et al., 2019a).

### DEG-miRNA interaction analysis

NetworkAnalyst (https://www.networkanalyst.ca/) is an online platform that aimed to provide a wide-range for meta-analyzing gene expression data and constructing gene regulatory networks in a user-friendly manner (Zhou et al., 2019b). The miRTarBase database provided comprehensive information on experimentally validated miRNA-target interactions and was used to identify regulatory miRNAs that influenced DEGs at the post-transcriptional level in NetworkAnalyst.

### Recognition of transcription factors

Transcription factors influence target genes at a transcriptional level. Using the Binding and Expression Target Analysis Minus algorithm, ENCODE targeted the transcription factor of genes derived from the ChIP-seq data. We adopted the ENCODE to predict regulatory TFs of our hub genes.

### Prediction of potential drugs of hub genes

The Drug–Gene Interaction Database (DGIdb) (http://www.dgidb.org/) is an online database for identifying drug-gene interaction by integrating the data from, for examplethe Drug Target Commons, DrugBank, TTD, PharmGKB, and Chembl and so on (Wagner et al., 2016). The common hub genes were imported into the database to search for potential drugs. The drug-gene interactions were visualized by the “ggalluvial” R package.

### Immune infiltration analysis

The enrichment for 28 immune infiltrating cells (Bindea et al., 2013) in the MDD and IS was assessed using a single-sample gene set enrichment analysis (ssGSEA) by using the “GSVA” R package (Hänzelmann et al., 2013). The immune cells with the same enrichment trends for both diseases and significant differences between diseases and the healthy controls were identified as the potential immune cells involved in the pathogenesis. The relationships between hub DEGs and immune cells were also constructed.

### Statistical analyses

R software (version R-4.1.0) performed all statistical analyses. The Wilcoxon test was used for statistical analysis between two groups. The relationships of genes with genes and genes with immune cells were constructed by using Pearson’s correlation method. A *p*-value less than 0.05 (*p* < 0.05) was considered to indicate statistical significance. The significance level is denoted as follows: *p < 0.05, **p < 0.01, and ***p < 0.001.

## Results

### Association between stroke and MDD

Baseline characteristics and the results of logistic regression analysis for stroke and MDD were shown in Table 1 and Table 2. The results indicated that MDD was significantly associated with an increased risk of stroke. Compared with non-exposure condition, the odds ratios (ORs) with 95% confidence intervals (CIs) for exposure condition between stroke and MDD was 2.79 (2.26,3.43), *p* < 0.0001.

**TABLE 1:** Baseline characteristics and odds ratio of participants by stroke levels in NHANES (2005–2018).

Variables	Stroke		*p*-value1	OR	95% CI	*p*-value2
	No	Yes				
MDD			<0.0001			
No	26,216 (92.69)	916 (81.98)		ref	ref	ref
Yes	2,366 (7.31)	208 (18.02)		2.79	2.79 (2.26,3.43)	<0.0001
Age (years)	46.85 (46.37,47.32)	63.46 (62.32,64.59)	<0.0001	1.07	1.07 (1.06,1.07)	<0.0001
Poverty	3.06 (3.00,3.13)	2.34 (2.21,2.47)	<0.0001	0.76	0.76 (0.72,0.80)	<0.0001
BMI(kg.m^2^)	28.99 (28.83,29.16)	30.07 (29.51,30.64)	<0.001	1.02	1.02 (1.01,1.03)	<0.001
Waist-circumference (cm)	99.17 (98.73, 99.61)	104.53 (103.12,105.94)	<0.0001	1.02	1.02 (1.01,1.02)	<0.0001
Sex			0.01			
Male	14,361 (49.61)	557 (44.51)		ref	ref	ref
Female	14,221 (50.39)	567 (55.49)		1.23	1.23 (1.05,1.43)	0.01
Race			<0.0001			
Non-Hispanic White	12,584 (69.21)	566 (70.84)		ref	ref	ref
Non-Hispanic Black	5,969 (10.48)	313 (14.75)		1.38	1.38 (1.19,1.59)	<0.0001
Mexican American	4,352 (8.03)	103 (4.51)		0.55	0.55 (0.43,0.70)	<0.0001
Other Hispanic	2,641 (5.20)	66 (2.83)		0.53	0.53 (0.38,0.74)	<0.001
Other	3,036 (7.08)	76 (7.07)		0.98	0.98 (0.69,1.38)	0.89
Education level			<0.0001			
Less than 9th grade	2,629 (4.57)	148 (8.44)		ref	ref	ref
9–11th grade	3,891 (10.01)	207 (15.23)		0.83	0.83 (0.63,1.09)	0.17
High school graduate	6,569 (23.11)	324 (31.87)		0.75	0.75 (0.60,0.93)	0.01
Some college	8,649 (31.94)	301 (27.00)		0.46	0.46 (0.36,0.58)	<0.0001
College graduate	6,844 (30.36)	144 (17.46)		0.31	0.31 (0.24,0.41)	<0.0001
Marital status			0.2			
Unmarried	13,730 (44.15)	582 (46.82)		ref	ref	ref
Married	14,852 (55.85)	542 (53.18)		0.9	0.90 (0.76,1.06)	0.20
Smoke			<0.0001			
Never	15,711 (54.93)	415 (38.18)		ref	ref	ref
Former	6,909 (24.78)	417 (35.86)		2.08	2.08 (1.76,2.46)	<0.0001
Now	5,962 (20.28)	292 (25.96)		1.84	1.84 (1.52,2.23)	<0.0001
Diabetes			<0.0001			
No	23,538 (86.80)	665 (63.27)		ref	ref	ref
Yes	5,044 (13.20)	459 (36.73)		3.82	3.82 (3.26,4.48)	<0.0001

**TABLE 2:** Baseline characteristics and odds ratio of participants by MDD levels in NHANES (2005–2018).

Variables	MDD		*p*-value1	OR	95% CI	*p*-value2
	No	Yes				
Stroke			<0.0001			
No	26,216 (97.53)	2,366 (93.41)		ref	ref	ref
Yes	916 (2.47)	208 (6.59)		2.79	2.79 (2.26,3.43)	<0.0001
Age (years)	47.37 (46.87,47.88)	46.54 (45.71,47.37)	0.08	1	1.00 (0.99,1.00)	0.08
Poverty	3.12 (3.05,3.18)	2.13 (2.02,2.24)	<0.0001	0.68	0.68 (0.65,0.71)	<0.0001
BMI(kg.m^2^)	28.90 (28.73,29.06)	30.54 (30.12,30.97)	<0.0001	1.03	1.03 (1.02,1.04)	<0.0001
Waist-circumference (cm)	99.06 (98.63, 99.49)	102.41 (101.36,103.47)	<0.0001	1.01	1.01 (1.01,1.02)	<0.0001
Sex			<0.0001			
Male	13,960 (50.56)	958 (36.20)		ref	ref	ref
Female	13,172 (49.44)	1,616 (63.80)		1.8	1.80 (1.62,2.01)	<0.0001
Race			<0.0001			
Non-Hispanic White	12,019 (69.60)	1,131 (65.15)		ref	ref	ref
Non-Hispanic Black	5,730 (10.41)	552 (12.85)		1.32	1.32 (1.16,1.50)	<0.0001
Mexican American	4,075 (7.96)	380 (7.60)		1.02	1.02 (0.85,1.22)	0.83
Other Hispanic	2,390 (4.96)	317 (7.19)		1.55	1.55 (1.27,1.89)	<0.0001
Other	2,918 (7.07)	194 (7.21)		1.09	1.09 (0.89,1.33)	0.41
Education level			<0.0001			
Less than 9th grade	2,428 (4.43)	349 (7.73)		ref	ref	ref
9–11th grade	3,568 (9.61)	530 (16.73)		0.96	0.96 (0.83,1.12)	0.64
High school graduate	6,259 (23.05)	634 (27.07)		0.65	0.65 (0.56,0.76)	<0.0001
Some college	8,167 (31.61)	783 (34.22)		0.61	0.61 (0.52,0.72)	<0.0001
College graduate	6,710 (31.30)	278 (14.25)		0.25	0.25 (0.20,0.32)	<0.0001
Marital status			<0.0001			
Unmarried	12,644 (42.72)	1,668 (62.43)		ref	ref	ref
Married	14,488 (57.28)	906 (37.57)		0.45	0.45 (0.40,0.50)	<0.0001
Smoke			<0.0001			
never	15,111 (55.83)	1,015 (37.94)		ref	ref	ref
former	6,743 (25.30)	583 (22.54)		1.31	1.31 (1.12,1.54)	0.001
now	5,278 (18.87)	976 (39.53)		3.08	3.08 (2.73,3.49)	<0.0001
Diabetes			<0.0001			
No	22,299 (86.69)	1904 (79.52)		ref	ref	ref
Yes	4,833 (13.31)	670 (20.48)		1.68	1.68 (1.48,1.90)	<0.0001

### Identification of DEGs and communal DEGs between MDD and IS

**FIGURE 1:**
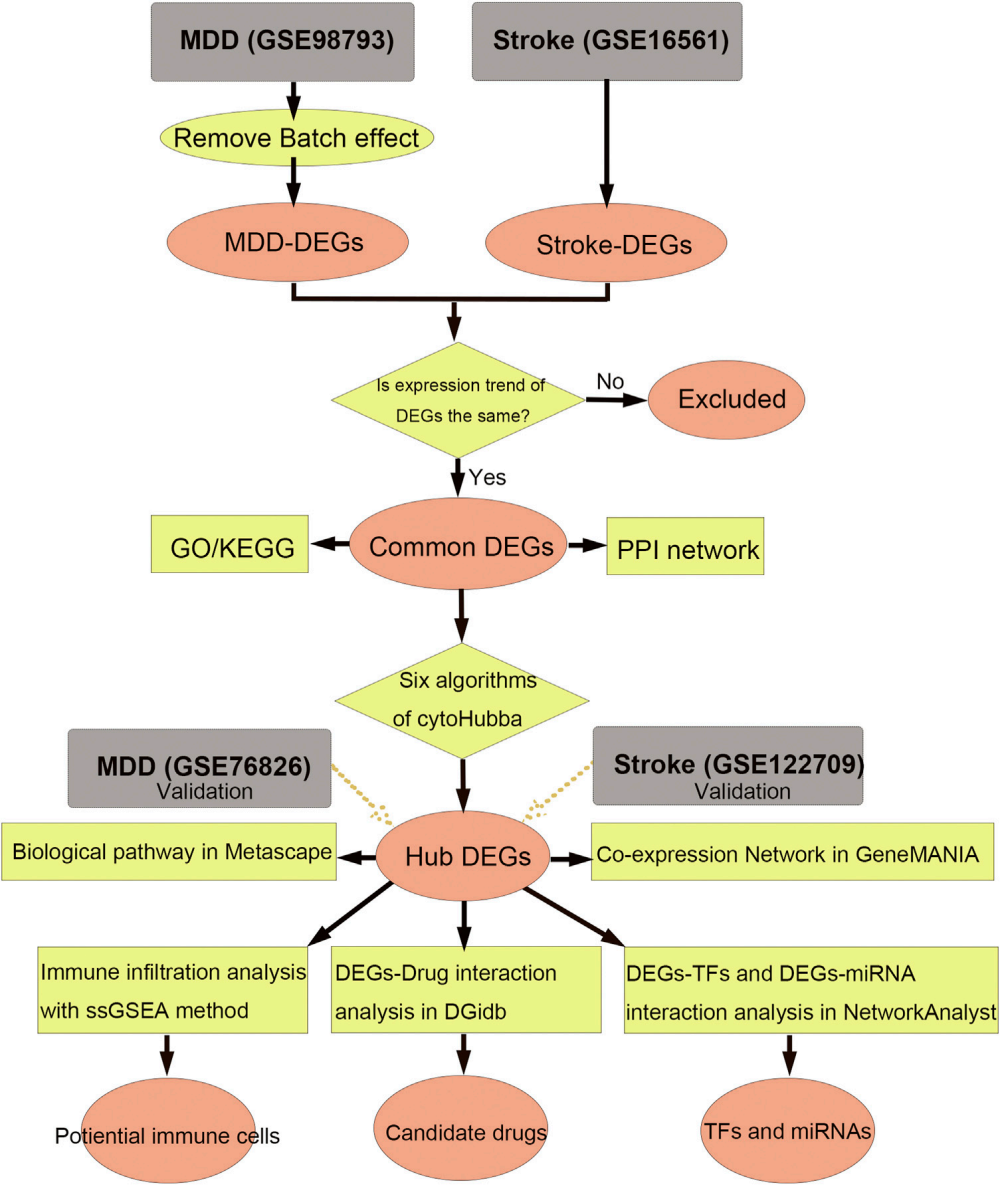
Workflow of data analysis in our present work. MDD, major depressive disorder; DEGs, differentially expressed genes; ssGSEA, single sample gene set enrichment analysis; TF, transcription factor; GO, Gene Ontology; KEGG, Kyoto Encyclopedia of Genes and Genomes; PPI, protein-protein interaction.

**FIGURE 2:**
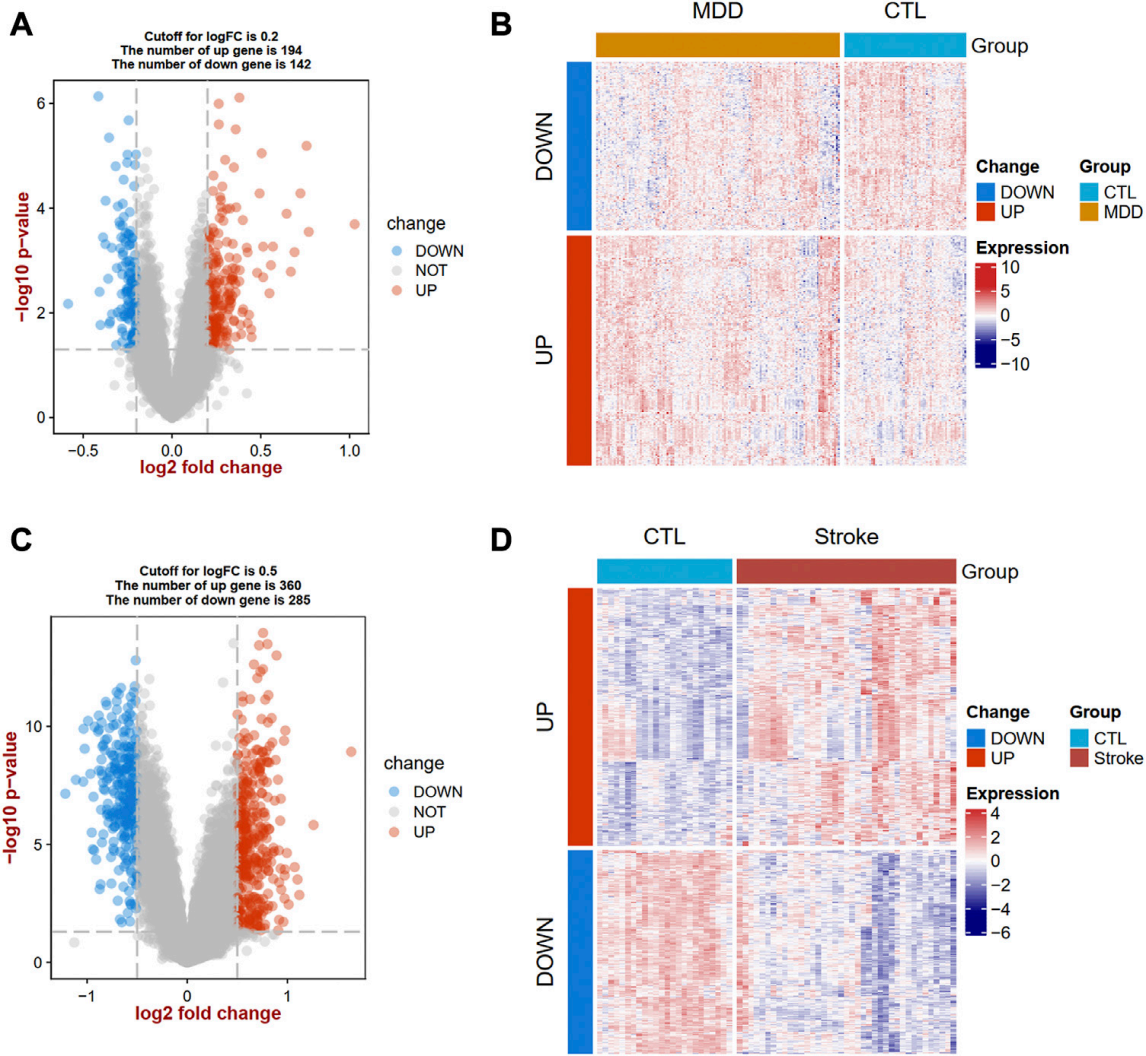
Landscapes of differentially expressed genes (DEGs) in MDD and IS. A volcano plot **(A)** and heat map **(B)** show the DEGs in MDD. A volcano plot **(C)** and heat map **(D)** show the DEGs in IS. MDD, major depressive disorder; IS, ischemic stroke.

The flow diagram for this study is shown in Figure 1. To uncover the interrelationships of IS with MDD, we first analyzed the human gene expression datasets from the GEO database to identify the dysregulated genes that stimulate MDD and IS separate. PCA results showed that there were two distinctive batches in GSE989793 in Supplementary Figure S1A, and the batch effect was removed in Supplementary Figure S1B. A volcano plot showed that a total of 336 DEGs were identified based on the following criteria: |log2FC|>0.2 and a *p*-value <0.05, including 194 that were upregulated and 142 that were downregulated between MDD patients and healthy controls in Figure 2A. These deregulated genes are shown with a heat map in Figure 2B. The GSE16561 dataset identified 360 upregulated and 295 downregulated genes taken from IS patient peripheral blood with the cut-off of |log2FC|>0.5 and a *p*-value <0.05. The deregulated genes were presented with a volcano and heat map plot in Figures 2C, D, respectively. The PCA result for IS patients and controls are shown in Supplementary Figure S2.

**FIGURE 3:**
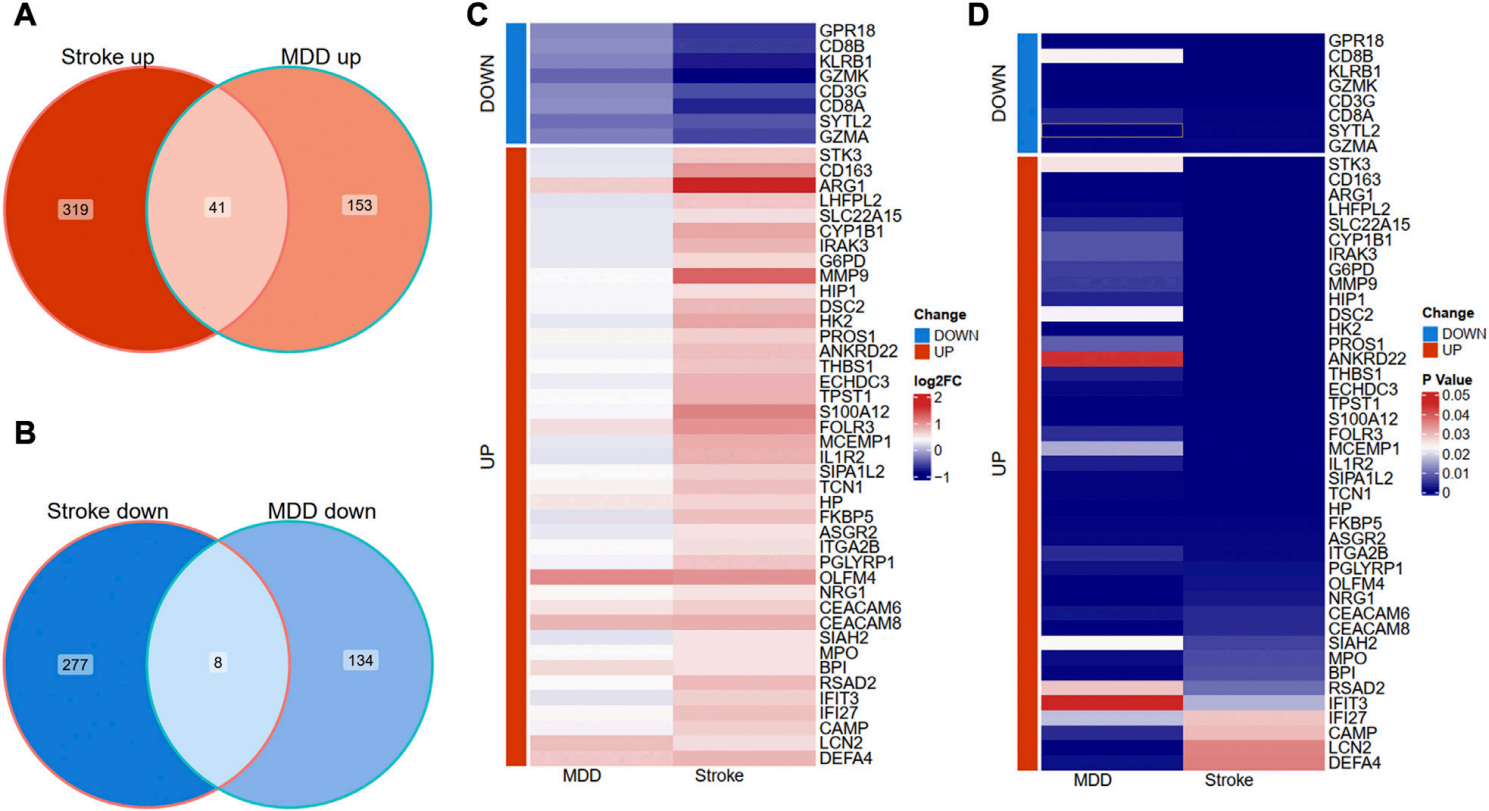
Identification of shared DEGs between MDD and IS. **(A)** Overlapping the shared upregulated DEGs. **(B)** Overlapping the shared downregulated DEGs. **(C)** A heat map indicates the shared DEGs from the perspective of log(Fold change). **(D)** A heat map indicates the shared DEGs from the perspective of the *p*-value. MDD, major depressive disorder; IS, ischemic stroke.

We further overlapped the deregulated genes of MDD and IS with the same expression trends. The Venn diagram showed that 41 common upregulated genes and eight common downregulated genes were finally identified in Figures 3A, B. The differential expression patterns in the two groups were presented with heat map plots from the perspective of logFC and *p*-value in Figures 3C, D, respectively.

### Functional enrichment analysis

**FIGURE 4:**
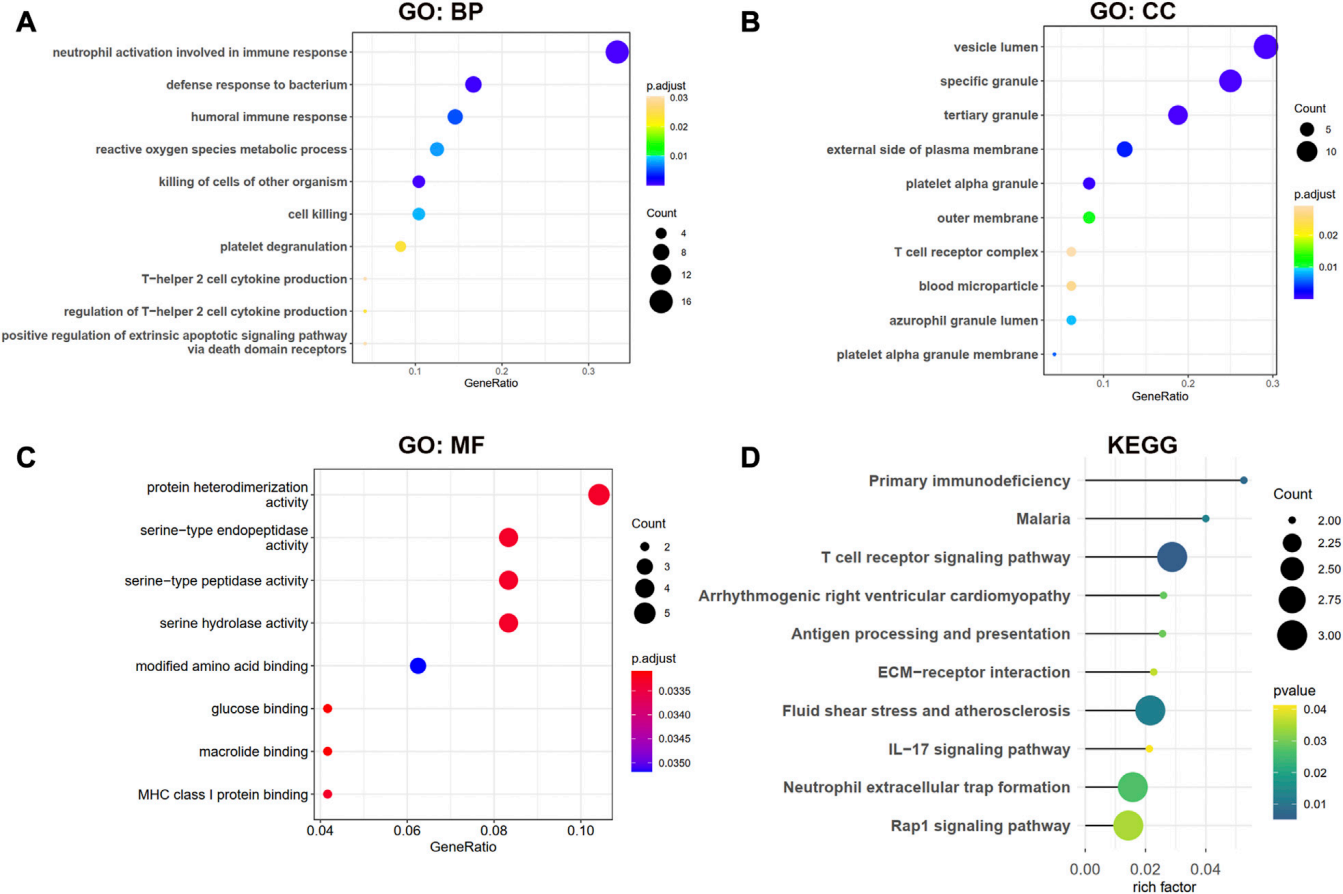
GO and KEGG enrichment analysis for shared DEGs between MDD and IS. **(A–C)** BP, CC, and MF of GO analysis were enriched for common DEGs. **(D)** KEGG pathways of common DEGs. BP, biological processes; CC, cellular components; MF, and molecular functions.

The biological processes (BP) results showed that the shared genes were mainly enriched in neutrophil activation involved in immune response, defense response to the bacterium, humoral immune response, and reactive oxygen species metabolic process (Figure 4A). In Figure 4B, we observed that these genes were involved in the vesicle lumen, specific granule, and tertiary granule cell component (CC). The molecular functions (MF) of these shared DEGs were enriched in protein heterodimerization activity and serine-related activity in Figure 4C. The KEGG result showed that the primary immunodeficiency, T cell receptor signaling pathway, and antigen processing and presentation pathways were enriched in Figure 4D.

### Identification and analysis of hub common DEGs

**FIGURE 5:**
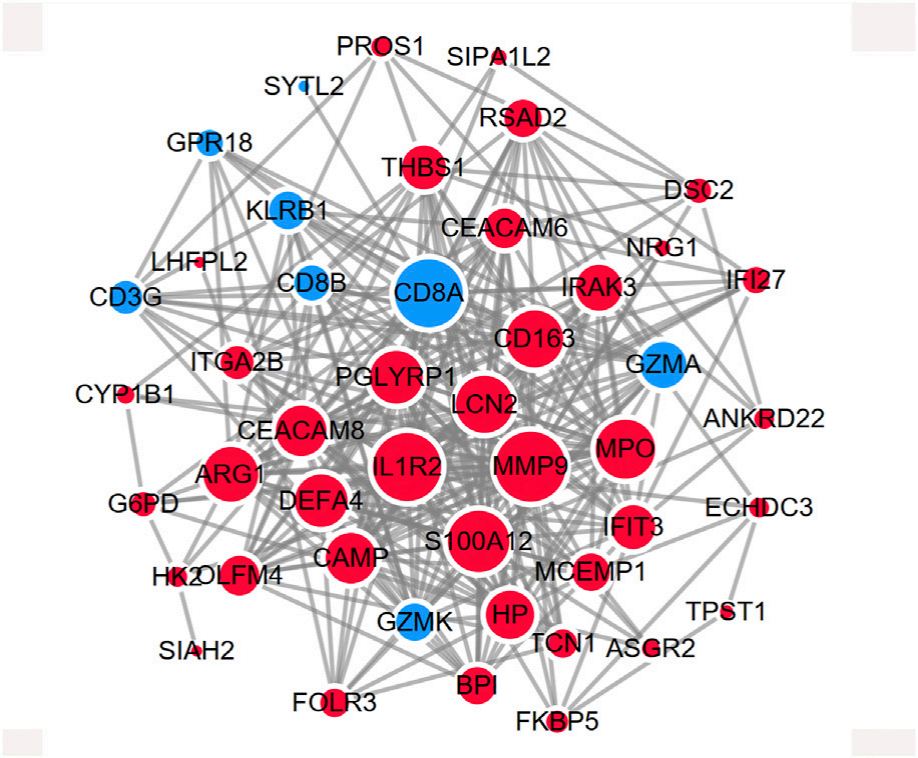
PPI network showing the protein interaction for shared DEGs between MDD and IS. The red color represents the commonly upregulated genes. The blue color represents the common downregulated genes. The size of the circle indicates the Degree of the node. The PPI network was generated using STRING and visualized in Cytoscape.

The shared genes were imported into the STRING database to construct a protein-protein network (Supplementary Figure S3). The network was further visualized in Cytoscape in Figure 5. The red represents the upregulated DEGs, while the turquoise represents the downregulated DEGs. The size of the node shows the degree of interaction with other genes.

**FIGURE 6:**
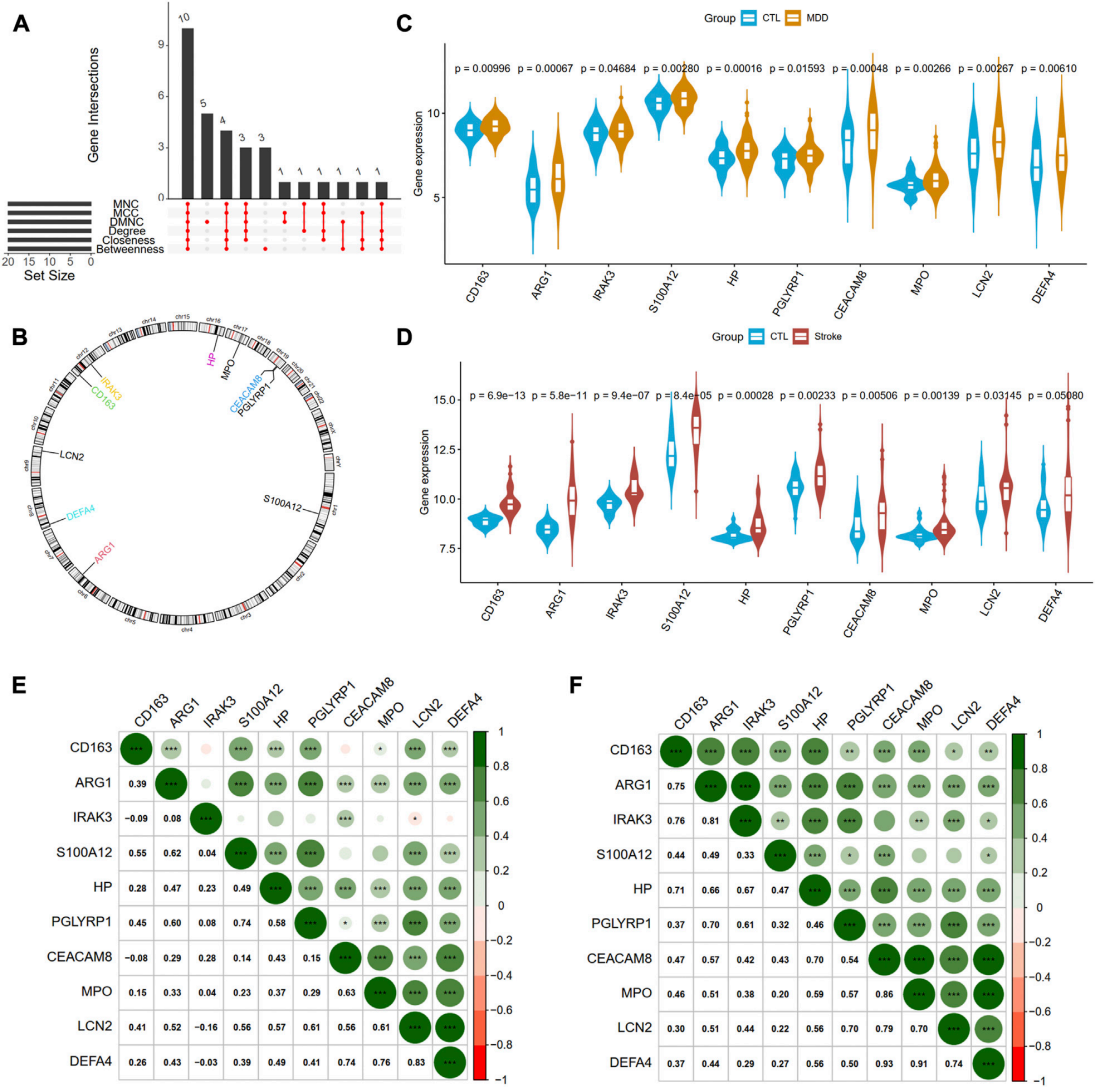
Identification of hub genes from PPI network. **(A)** A Venn diagram shows ten overlapped genes that were screened out by the six methods from the top 20 genes of six methods in Cytohubba plug in Cytoscape. **(B)** The gene locations of the ten hub genes. **(C)** and **(D)** shows the expression levels of the ten hub genes in MDD and IS, respectively. **(E)** and **(F)** depicted the correlations of the ten genes with each other in MDD and IS, respectively. Significance level was denoted by *p‐value <.05, **p‐value <.01, ***p‐value <.001.

Next, we adopted six algorithms in the cytoHubb plugin of Cytoscape to identify the hub genes. The top 20 genes in each method were visualized in Supplementary Figure S4 and listed in Table 3. We then intersected the 20 genes for each method, and ten overlapped genes (CD163, AEG1, IRAK3, S100A12, HP, PGLYRP1, CEACAM8, MPO, LCN2, and DEFA4) denominated as hub communal DEGs were selected in Figure 6A. The detailed descriptions of the hub genes were listed in Table 4, and that of other DEGs were in Supplementary Table S1. The locations of the 10 genes in the corresponding chromosome are presented in Figure 6B. The violin plot showed that the hub shared genes were significantly expressed in MDD and IS with the same trend in Figures 6C, D. The relationships among genes show that most genes were significantly positively related to each other in MDD (Figure 6E) and stroke (Figure 6F).

**TABLE 3:** The top20 genes identified by six different methods.

Rank	MNC	MCC	DMNC	Degree	Closeness	Betweenness
1	MMP9	MMP9	BPI	MMP9	MMP9	MMP9
2	IL1R2	S100A12	CAMP	IL1R2	IL1R2	CD8A
3	CD8A	MPO	OLFM4	CD8A	CD8A	IL1R2
4	S100A12	LCN2	LCN2	S100A12	S100A12	ITGA2B
5	MPO	CAMP	CEACAM8	MPO	MPO	S100A12
6	LCN2	ARG1	PGLYRP1	LCN2	LCN2	HK2
7	CD163	CD8A	TCN1	CD163	CD163	THBS1
8	ARG1	PGLYRP1	IRAK3	ARG1	ARG1	ARG1
9	PGLYRP1	HP	HP	PGLYRP1	PGLYRP1	DEFA4
10	CEACAM8	IL1R2	MPO	DEFA4	DEFA4	IRAK3
11	CAMP	CEACAM8	CD163	CEACAM8	CEACAM8	MPO
12	DEFA4	CD163	KLRB1	CAMP	CAMP	FKBP5
13	HP	BPI	S100A12	HP	HP	LCN2
14	GZMA	DEFA4	ARG1	IRAK3	IRAK3	CD163
15	IRAK3	IRAK3	DEFA4	GZMA	GZMA	HP
16	IFIT3	OLFM4	GZMA	IFIT3	THBS1	IFIT3
17	THBS1	THBS1	IFI27	THBS1	IFIT3	MCEMP1
18	CEACAM6	GZMA	GZMK	CEACAM6	CEACAM6	PGLYRP1
19	OLFM4	CEACAM6	CD3G	OLFM4	OLFM4	CEACAM8
20	RSAD2	MCEMP1	ITGA2B	RSAD2	MCEMP1	ECHDC3

**TABLE 4:** The detailed information and descriptions of hub genes.

Gene name	Ensembl id	Gene description	Chromosome	Change
CD163	ENSG00000177575	CD163 molecule	12	UP
ARG1	ENSG00000118520	arginase 1	6	UP
IRAK3	ENSG00000090376	interleukin 1 receptor associated kinase 3	12	UP
S100A12	ENSG00000163221	S100 calcium binding protein A12	1	UP
HP	ENSG00000257017	haptoglobin	16	UP
PGLYRP1	ENSG00000008438	peptidoglycan recognition protein 1	19	UP
CEACAM8	ENSG00000124469	CEA cell adhesion molecule 8	19	UP
MPO	ENSG00000005381	myeloperoxidase	17	UP
LCN2	ENSG00000148346	lipocalin 2	9	UP
DEFA4	ENSG00000285318	defensin alpha 4	8	UP

**FIGURE 7:**
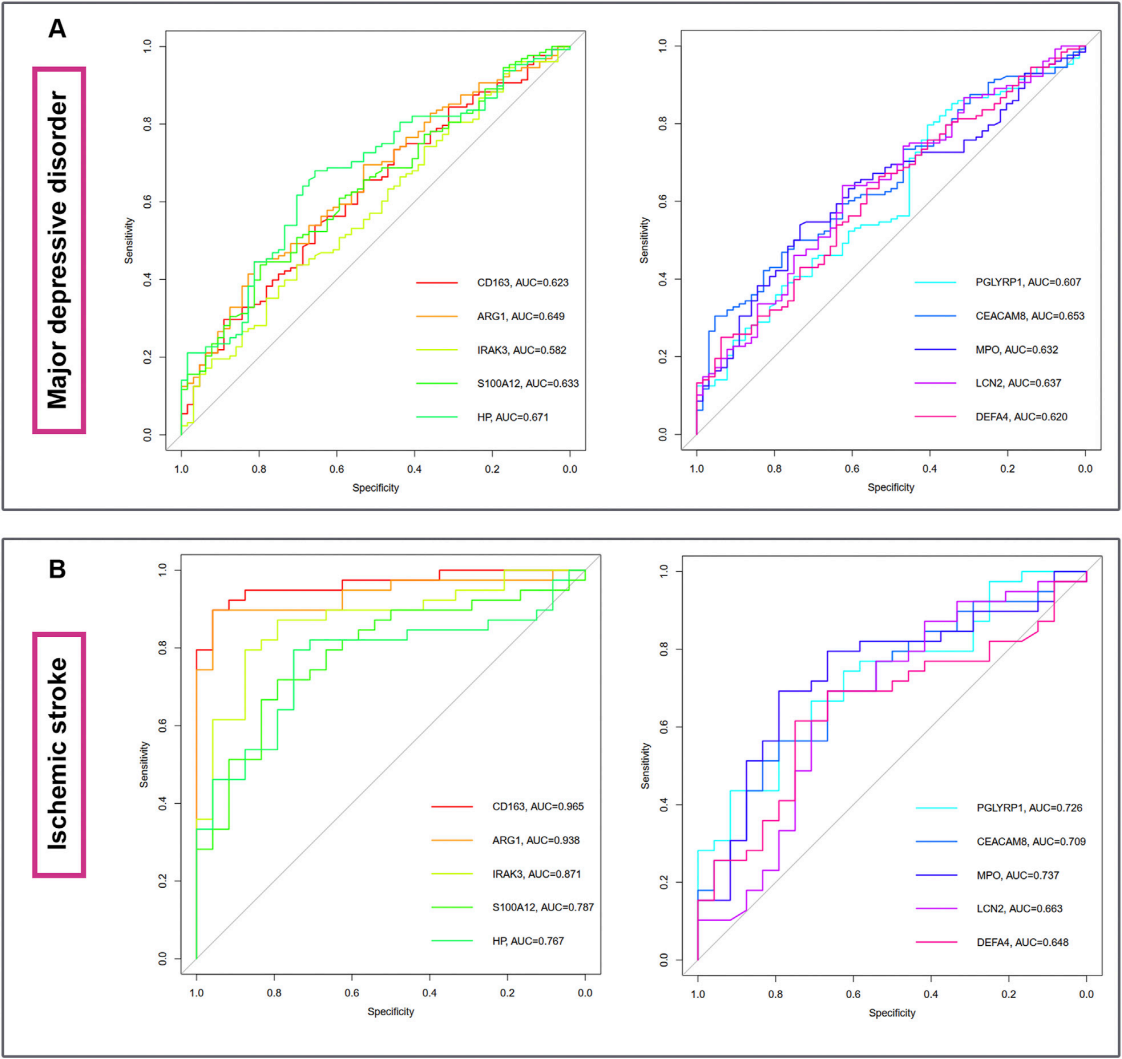
The diagnostic abilities of the ten hub genes in MDD **(A)** and IS **(B)** with ROC curve. ROC, receiver operating characteristic.

The diagnostic ablility of the hub genes in MDD (Figure 7A) and IS (Figure 7B) were visualized with receiver operating characteristic curves. The results shows that HP present the greatest diagnostic value with AUC = 0.671 in MDD, while CD163 display the greatest diagnostic value with AUC = 0.965 in IS.

We further validated the epression of the ten hub genes in other external datasets (Supplementary Figure S5). However, only S100A12 was validated as the common differentially expressed gene of the two disorders, which need to be verified by *in vivo* or vitro experiments.

**FIGURE 8:**
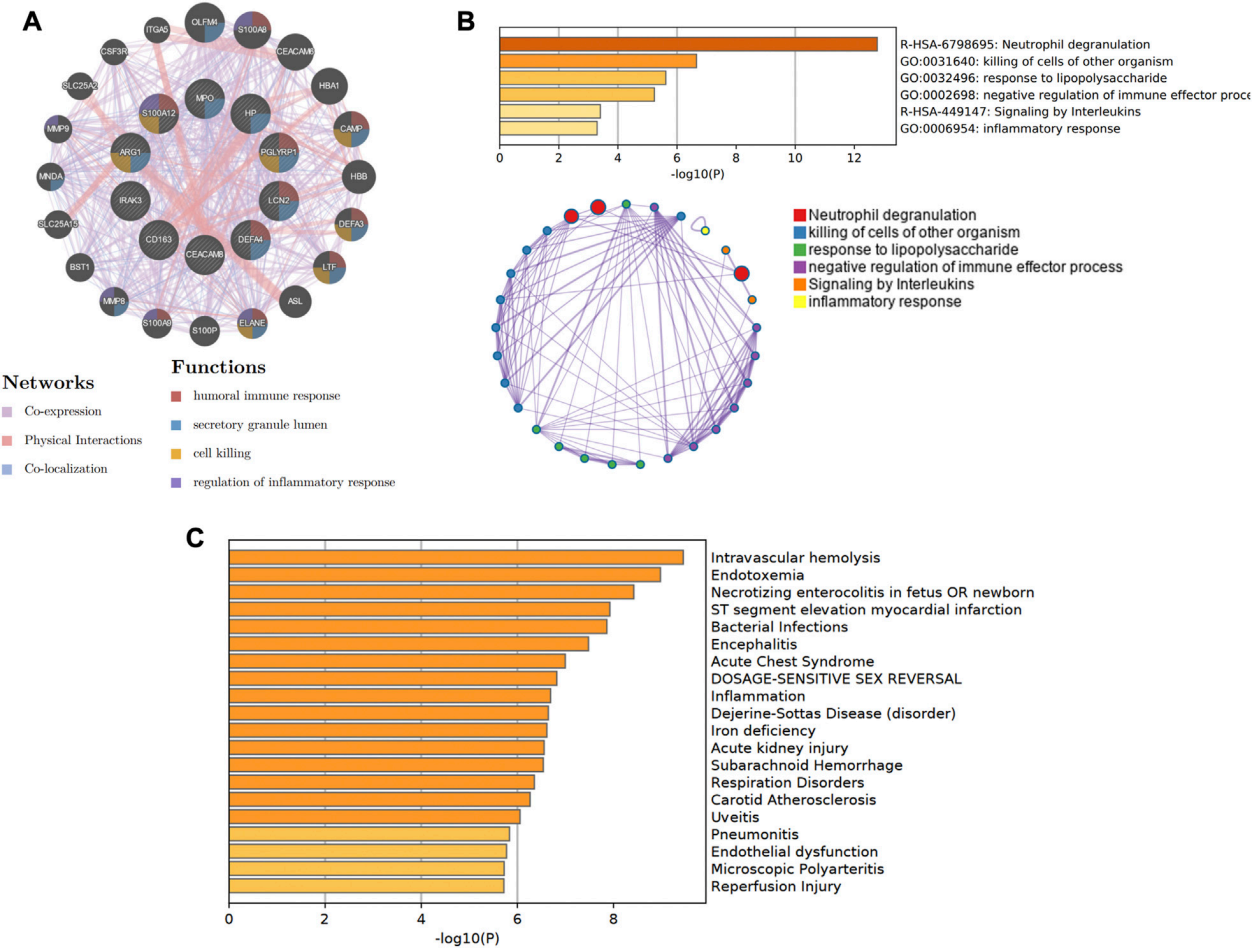
The co-expression network and biological functions of hub genes. **(A)** Hub genes and their co-expression genes were analyzed *via* GeneMANIA. **(B)** The biological pathways were enriched for hub genes *via* Metascape. **(C)** The potential diseases were participated by these hub genes from the DisGeNET database.

In Figure 8A, the co-expression network of hub genes was constructed using the GeneMANIA website. In the complex PPI network, the interaction of the co-expression accounted for 74.83%, physical interactions for 22.14%, and colocalization for 3.04%. In function analysis, these genes were involved in humoral immune response, secretory granule lumen, cell killing, and regulation of inflammatory response, which was almost consistent with the results from the Metascape analysis (Figure 8B; Supplementary Table S2). In Figure 8C and Supplementary Table S3, we also predicted the potential diseases that the hub genes may be involved in through gene-disease association information collected from the DisGeNET database (Piñero et al., 2020) in Metascape. The results showed that these genes participate in intravascular hemolysis, endotoxemia, and bacterial infections.

### Gene regulatory network analysis of Genes-miRNAs and Genes-TFs

**FIGURE 9:**
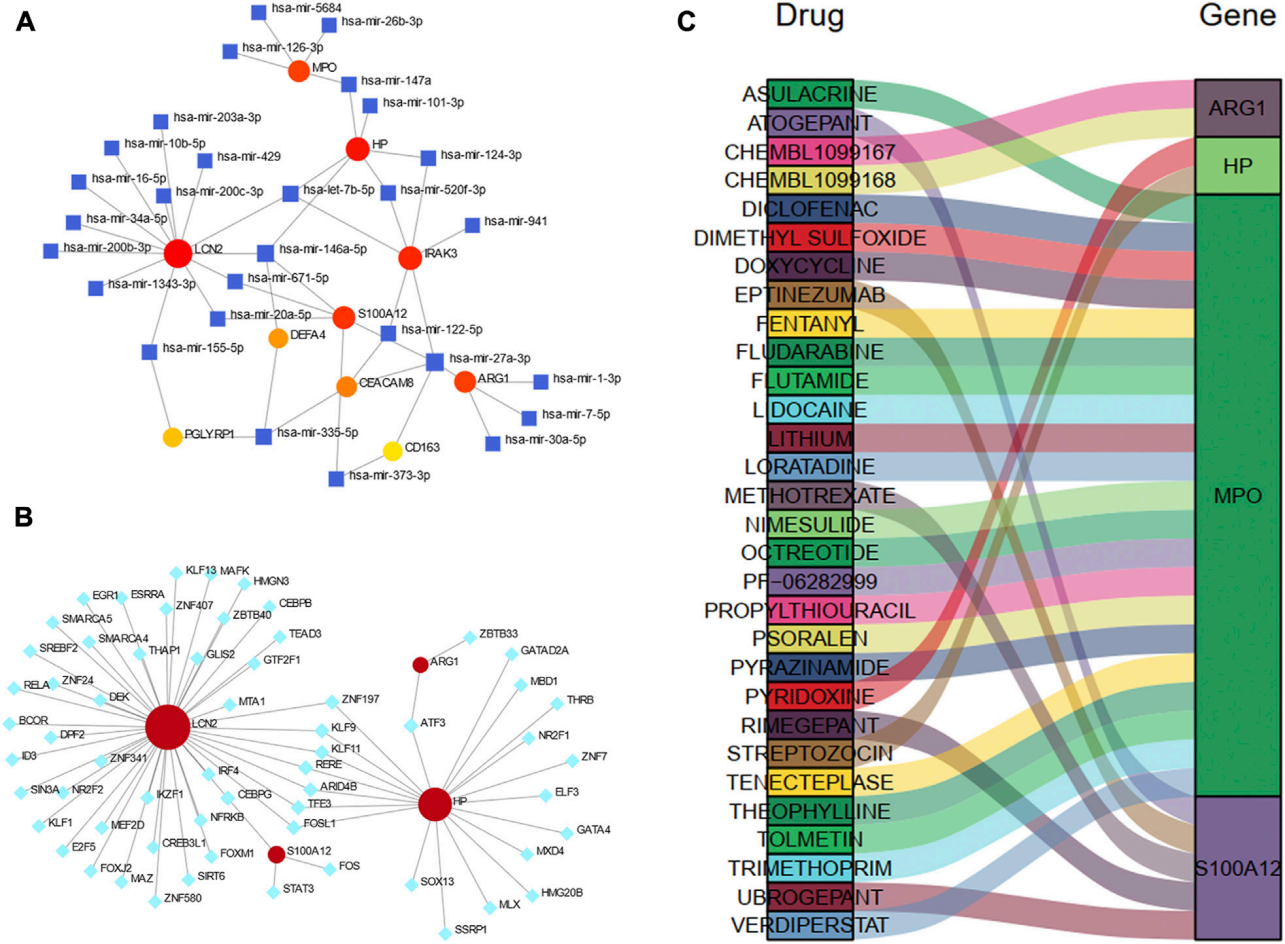
Construction of the regulatory network for hub genes. **(A)** gene-miRNAs interactions. **(B)** gene-TFs interactions. **(C)** A Sankey diagram indicating the potential drugs for hub genes. The circle nodes represent the hub genes. The square nodes represent miRNAs. The diamond nodes represent TFs. TF, transcription factor.

TarBase database was utilized to predict the miRNA of hub genes. All hub shared genes were predicted for their interacted miRNA, and a total of 28 miRNA were determined in Figure 9A. In the gene-miRNA interaction network, LCN2 interacted with the most miRNAs with 13 predicted, followed by HP and IRAK3 with 6 miRNAs. hsa-mir-27a-3p were located in a conspicuous place due to interacting with five hub genes.

We also predicted the experimentally validated TFs of hub genes using ENCODE database in Figure 9B. Only four hub genes predicted their regulatory TFs. A total of 61 TFs were identified, and LCN2 also had the most targeting nodes with 45 TFs, followed by HP with 20 TFs.

The Sankey diagram showed the potential drugs that targeted the hub genes from the DGIdb database (Figure 9C). A total of 30 drugs were predicted, and the detailed information were listed in Supplementary Table S4. Of these, 21 drugs targeted MPO; five drugs targeted S100A12, and 2 drugs each targeted HP and ARG1. No potential drugs could be identified for LCN2, DEFA4, PGLYRP1, CEACAM8, CS163, and IRAK3.

### Immune cell infiltration analysis

**FIGURE 10:**
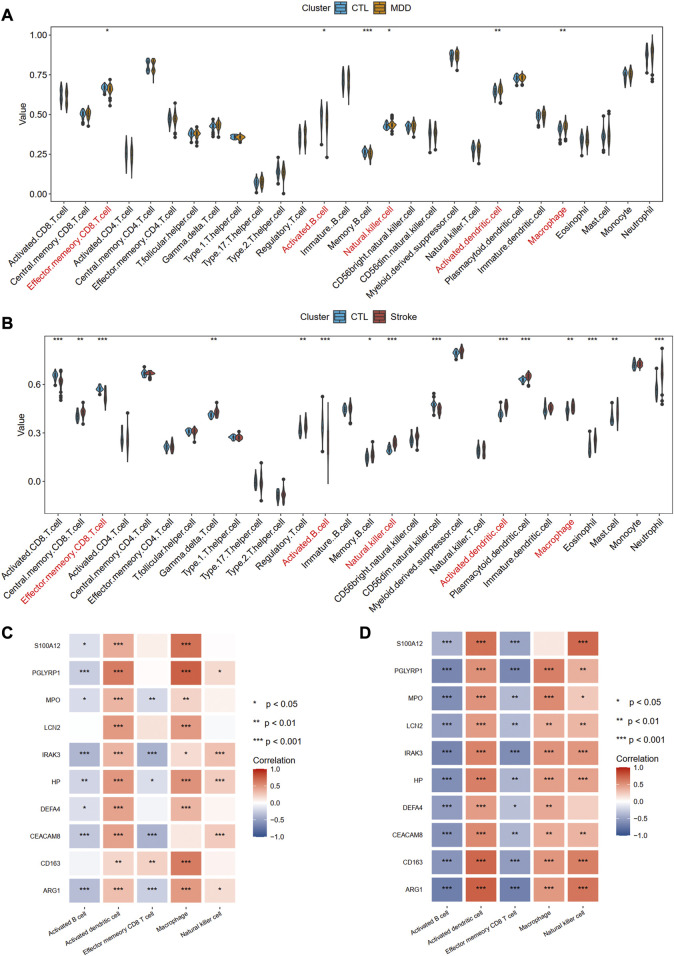
Identification of common immune cells between MDD and IS. **(A)** The abundance of the immune cell in MDD using the ssGSEA method. **(B)** The abundance of the immune cell in IS using the ssGSEA method. The immune cells with red color indicate the significantly common immune cells. **(C)** and **(D)** A heat map visualized the correlations between common immune cells and shared hub genes. Significance level was denoted by *p‐value <.05, **p‐value <.01, ***p‐value <.001.

Using the ssGSEA algorithm, we obtained the immune infiltration of 28 immune cells in the MDD group, IS group, and control group. The immune cells with significant differences between cases and the healthy control group and the same trends were regarded as the potential cells. A total of five immune cells among these 28 types of cells, including activated B cell, activated dendritic cell, effector memory CD8 T cell, macrophage, and natural killer cell were identified, among which active B cell and effector memory CD8 T cell were downregulated, whereas other cells were upregulated, implying the innate immunity was activated while acquired immunity was suppressed in the two diseases (Figures 10A, B). Figures 10C, D also showed the strong relationships between the hub gene and immune cells.

## Discussion

Depression is a global health problem with a high prevalence and the third leading cause of disability globally (Park and Zarate, 2019). The incidence of suicide associated with depression has been increasing and is the 10th leading cause of death in the United States Similarly, stroke, a neurological disorder characterized by blockage of blood vessels, is a major cause of death and disability worldwide (Johnston et al., 2009). Early studies indicated that depression increased the risk of stroke. A prospective longitudinal study showed that a history of depression was associated with an increased risk of stroke by over twofold (Jackson and Mishra, 2013). Compared with participants with stable low/no depressive symptoms, the participants with the stable high and remitted depressive symptoms had a 2.14 and 1.66 elevated hazard risk of stroke, respectively (Gilsanz et al., 2015). In addtion, post-stroke depression (PSD) is one of the common and serious sequelae of stroke. Folstein et al. (1977) first demonstrated that mood disorder is a more specific complication of stroke. Disability, anxiety, stroke severity, depression pre-stroke, and cognitive impairment all play an important role in PSD, according to a meta-analysis (Ayerbe et al., 2013). Although the bi-directional relationship between stroke and depression is recognized, the underlying mechanism remains a provocative and unresolved question. Considering that stroke and depression have genetic roots, as well as their frequent comorbidity, we speculate shared genes and biological pathways for both stroke and depression.

With the rapid development of sequencing technology, for the first time, we explored the shared gene signatures and molecular mechanisms between MDD and IS from transcriptome data. In our study, we observe a total of 41 genes are simultaneously upregulated, and eight genes are downregulated in both MDD and IS. Biological enrichment analysis shows that these common genes are involved in the immune response, cell killing, and defense response to the bacterium. Moreover, the T cell receptor signaling pathway, primary immunodeficiency, malaria, IL-17 signaling pathway, and rap1 signaling pathway are enriched in the KEGG pathway analysis. Ten overlapped genes (CD163, AEG1, IRAK3, S100A12, HP, PGLYRP1, CEACAM8, MPO, LCN2, and DEFA4) denominated as hub communal DEGs are identified. We observe that the ten hub genes participate in the immune response and cell killing processes. The gene-diseases analysis reveals that intravascular hemolysis, endotoxemia, and bacterial infections are correlated with these genes. Furthermore, we construct gene regulatory networks with gene-miRNA, gene-TF, and gene-drugs, which further provide targets for therapeutic interventions. Finally, we depict the immune landscapes for both disorders and found that five immune cells, including activated B cell, activated dendritic cell, effector memory CD8 T cell, macrophage, and natural killer cell, were significantly different in both diseases. Further analysis indicates innate immunity may be activated whereas acquired immunity may be suppressed.

There is consistently and robust evidence supporting the role of inflammation in depression. The inflammatory response in MDD patients was characterized by increased production of complement, chemoattractors, and pro-inflammatory cytokines in peripheral blood cerebrospinal fluid, and post-mortem brain samples (Miller and Raison, 2016). Cytokines, which mediate the innate immune response, including IL-1, tumor necrosis factor (TNF)-alpha, C-reactive protein (CRP), and IL-6, from peripheral blood are considered the most reliable biomarkers of inflammation in patients with depression (Miller et al., 2009). In addition, by inhibiting pro-inflammatory cytokines or their signaling pathways, depressed mood can be improved and conventional antidepressants better tolerated (Kenis and Maes, 2002; Bluthé et al., 2006). Furthermore, by producing anti-inflammatory cytokines (IL-2, IL-4, and IL-10) and/or activating T regulatory (Treg) cells, the effects of immune response were also counter-balanced or compensated in MDD patients (Dowlati et al., 2010). Growing evidence also revealed an intimate relationship between the immune system and all stages of the ischemic cascade, from the acute intravascular events induced by a blockage of the blood supply to the parenchymal process causing brain damage (Iadecola and Anrather, 2011; Endres et al., 2022). A recent review summarized that pro-inflammatory interleukins (IL-1b, IL-6, IL-8, IL-12, IL-15, IL-16, IL-20, IL-18, and IL-23/IL-17) and anti-inflammatory interleukins (IL-2, IL-4, IL-10, IL-13, IL-19, and IL-33) were involved in the pathogenesis of IS (Zhu et al., 2022). As inflammation is common after stroke and depression, immunological processes were proposed as the underlying mechanism triggering PSD (Pascoe et al., 2011). Inflammatory markers such as CRP, ferritin, and neopterin have been linked to PSD development later in life (Becker, 2016). Our GO and KEGG analysis demonstrates that the immune response is enriched in the common DEGs and the ten hub genes of MDD and IS. Moreover, in our immune infiltration analysis, the abundance of active B cells and effector memory CD8 T cell decreases, while that of activated dendritic cell, macrophage, and natural killer cell increases in both disorders, which may provide new insight into the common pathogenesis and immunotherapy for both diseases. Specifically, the IL-17 signaling pathway was also observed in our biological function annotation of shared genes. The IL-17 family is an evolutionarily old cytokine family consisting of six members (IL-17A-F), dominantly produced by immune cells of the adaptive and innate lymphocyte lineages, including CD4^+^ Th17 cells, CD8^+^ Tc17 cells, γδT17 cells, MAIT cells, and innate lymphoid cells ILC3 (Majumder and McGeachy, 2021). It has been observed that IL-17 levels are high in the central nervous system (CNS) during inflammatory responses, including IS and MDD. Peripheral blood samples from patients with IS show an increased expression of IL-17 compared with healthy individuals (Kostulas et al., 1999). High plasma levels of IL-17 were also detected in MDD patients (Waisman et al., 2015). Combined with our results, we speculate that the IL-17 signaling pathway plays an important role in the shared mechanisms of MDD and IS.

A PPI network analysis was conducted among the proteins derived from shared DEGs to depict functional and physical interactions between IS and MDD. By integrating with six algorithms (MCC, MNC, DMNC, Degree, Closeness, and Betweenness) in the cytoHubba plugin of Cytoscape, we identify ten hub communal DEGs (CD163, AEG1, IRAK3, S100A12, HP, PGLYRP1, CEACAM8, MPO, LCN2, and DEFA4), which may serve as potential interventional targets.

CD163, the hemoglobin scavenger receptor, is a macrophage-specific protein of the “alternative activation” phenotype and played a major role in dampening the inflammatory response (Moestrup and Møller, 2004). The upregulation of CD163 in monocytes was observed in MDD patients compared with healthy controls (Simon et al., 2021). However, in MDD *in vitro* experiments, sub-anesthetic doses of ketamine, an antidepressant (Murrough et al., 2013), program human monocytes into M2c-like macrophages (anti-inflammatory phenotype) by inducing high levels of CD163 and MERTK (Nowak et al., 2019). Compared with the CD14^+^ classical subtype, CD163 expression was more pronounced in CD16^+^ non-classical and intermediate monocytes after IS and may serve as a potential biomarker of monocyte activation (Greco et al., 2021). Moreover, the percentage of CD163+/CD16+ events 24 h after IS was positively associated with stroke severity and disability. In our analysis, in comparison with the controls, the higher expression of CD163 in both MDD and IS is observed, which may act as a shared risk gene for IS and MDD.

The interleukin receptor-associated kinase (IRAK) family [including IRAK-1, IRAK-2, IRAK-M (IRAK-3), and IRAK-4] are involved in regulating Toll-like receptor (TLR) and interleukin-1 (IL-1) signaling pathways. Interleukin one receptor-associated kinase 3 (IRAK3) is a protein of 596 amino acids with a molecular mass of 68 kDa and is limited to monocytes and macrophages (Wesche et al., 1999). A recent genome-wide association study (GWAS) identified a genome-wide significant locus (rs11465988) in IRAK3 for esketamine efficacy of anti-depression (i.e., percentage change in symptom severity score compared with baseline). The potential roles of IRAK3 in IS have also been discovered recently. The expression levels of IRAK3 that may link natural killer cells to apoptosis were upregulated in IS through bioinformatics analysis (Feng et al., 2022). In experimental stroke mice, IRAK3 has neuroprotective effects, and its deletion can exacerbate neurovascular damages (Lyu et al., 2018). However, our results identify the enhanced expression in both IS and MDD.

Myeloperoxidase (MPO) is a member of the superfamily of heme peroxidases, that is, mainly found in neutrophils and monocytes. High levels of MPO have been detected in the serum of depressive patients in a twin study (Vaccarino et al., 2008). Inhibiting MPO activity and serotonin reuptake may be a potential new approach to MDD with inflammatory syndrome (Soubhye et al., 2014). Moreover, a significant increase in MPO mRNA expression was observed in peripheral blood cells from patients with recurrent depressive disorder (rDD) compared to controls (Gałecki et al., 2012; Talarowska et al., 2015). The expression of MPO was also associated with the risk of IS (Wright et al., 2009). Concentrations of serum MPO are increased after IS and was associated with stroke severity (Palm et al., 2018; Orion et al., 2020). Inhibiting MPO activity increased cell proliferation and improved neurogenesis after IS (Kim et al., 2016; Kim et al., 2019). Although MPO contributes to both IS and MDD, few studies were conducted to explore the potential mechanism of IS complicated with MDD. Our result may provide a bridge linking the two disorders.

Lipocalin-2 (LCN2) is a member of the highly heterogeneous lipocalin family of secretory proteins. The roles of LCN2 in IS and depression have been proved recently (Zhao et al., 2019b; Vichaya et al., 2019). A study demonstrated that the relationship between LCN2 and the process of PSD may be mediated *via* the P38 MAPK pathway (Wei et al., 2021). Our study provides potential association for LCN2 and comorbidity between depression and IS.

Haptoglobin encoded by HP participates in the process of depression and stroke from the perspective of genetic and proteomic levels (Maes et al., 1993; Kiga et al., 2008; Ijäs et al., 2013). Considering few studies focusing on the intermediate role of haptoglobin between depression and stroke, this study provides new insight and reference for investigating its potential roles in the comorbidity, such as PSD.

Regulatory biomolecules may serve as potential interventional targets in multiple complex illnesses. TFs play a key role in regulating the ratio of transcription, and miRNAs handle gene regulation and RNA silencing at the post-transcription level. Given the crucial roles of the ten hub common genes, we also analyze the TFs–gene, miRNAs-gene, and drugs-gene interaction to find transcriptional, post-transcriptional, and therapeutic regulators. mir-27a-3p, mir-146a-5p, mir-335-5p, and let-7b-5p are identified to be interacting with at least three hub genes. Furthermore, we discovered that TFs (such as ZNF197, KLF9, KLF11, RERE, ARID4B, TFE3, and FOSL1) target LCN2 and HP simultaneously. Finally, 31 candidate drugs were predicted, among which 21 drugs target MPO. Combined with the above-mentioned roles of MPO in both depression and stroke, these drugs may serve as potential theraputics to treat the comorbidities.

Some limitations should be noted in our work. First, although the gene expression profiling from both diseases are derived from the same tissues, there is inadequate information regarding the blood sample collection time for the studies. The disease course of depression and stroke are different. Second, in this study, all the results were acquired by bioinformatic analysis, and we have not conducted any *in vivo* or *in vitro* experiments to verify the different expression levels. Hence, the findings should be interpreted with caution.

## Conclusion

We performed a bioinformatic analysis to identify overlapping DEGs subserving both MDD and IS. The communal DEGs participate in the immune response and cell killing processes. Furthermore, ten hub DEGs (CD163, AEG1, IRAK3, S100A12, HP, PGLYRP1, CEACAM8, MPO, LCN2, and DEFA4) were screened out based on six algorithms (MCC, MNC, DMNC, Degree, Closeness, and Betweenness). Immune infiltration analysis shows that the innate immunity was activated whereas acquired immunity was suppressed in both diseases. These findings increase our understating of the association of IS with depression at a transcriptional level. The final gene regulatory network may shed light on novel therapeutic targets for both disorders.

## Data Availability

Publicly available datasets were analyzed in this study. The names of the repository/repositories and accession number(s) can be found in the article/Supplementary Material.
