# Corrigendum: Exploring the bi-directional relationship and shared genes between depression and stroke via NHANES and bioinformatic analysis

**DOI:** 10.3389/fgene.2024.1405696

**Published:** 2024-04-10

**Authors:** Zhanghuan Yang, Maokun He, Qian Zhang, Shifu Li, Hua Chen, Di Liao

**Affiliations:** ^1^ Department of Oncology, Xiangya Cancer Center, Xiangya Hospital, Central South University, Changsha, Hunan, China; ^2^ Hainan Medical University, Haikou, Hainan, China; ^3^ Department of Neurosurgery, Xiangya Hospital, Central South University, Changsha, Hunan, China; ^4^ National Clinical Research Center for Geriatric Disorders, Central South University, Changsha, Hunan, China; ^5^ Department of Neurosurgery, The First people’s Hospital of Changde, Changde, China; ^6^ Department of Neurology, Xiangya Hospital, Central South University, Changsha, Hunan, China

**Keywords:** ischemic stroke, major depressive disorder, bioinformatics, shared genes, immune infiltration, NHANES

In the published article, there was an error in the **Funding** statement. The correct **Funding** statement appears below.

