# Supplementary material for: Exploring the bi-directional relationship and shared genes between depression and stroke via NHANES and bioinformatic analysis
Source: Front Genet. 2023 Mar 31;14:1004457. doi: 10.3389/fgene.2023.1004457 (PMC10102600; doi:10.3389/fgene.2023.1004457)
Supplement: Supplementary file 1 [file DataSheet1.docx]

Supplementary materials


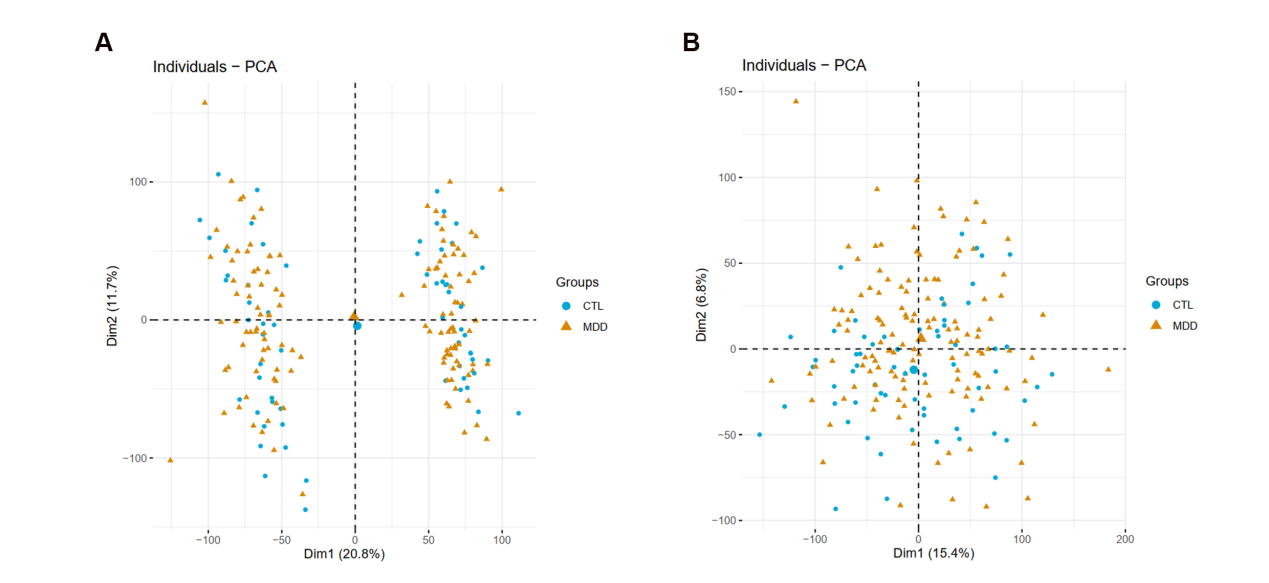


Figure S1. The principal component analysis of GSE98793 dataset before (A) and after (B) removing batch effect.


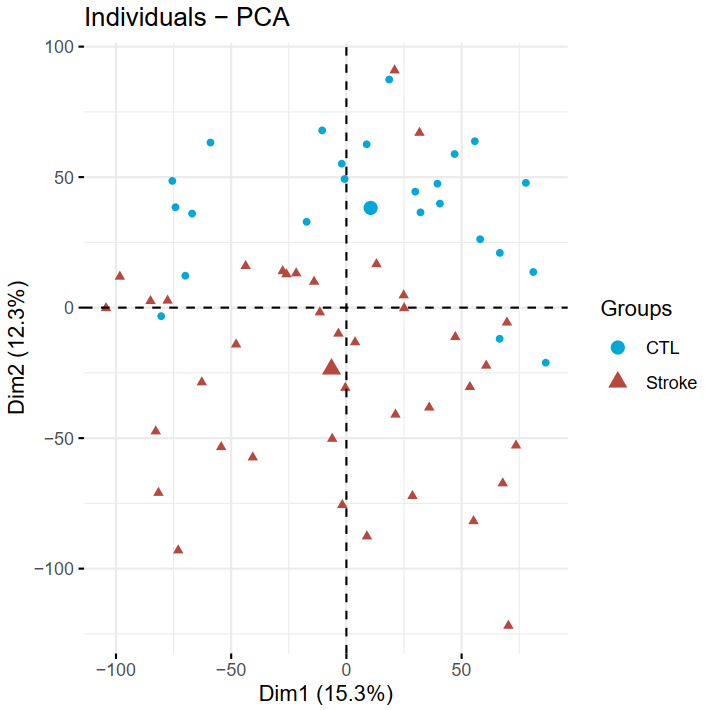


Figure S2. The principal component analysis of GSE16561 dataset.
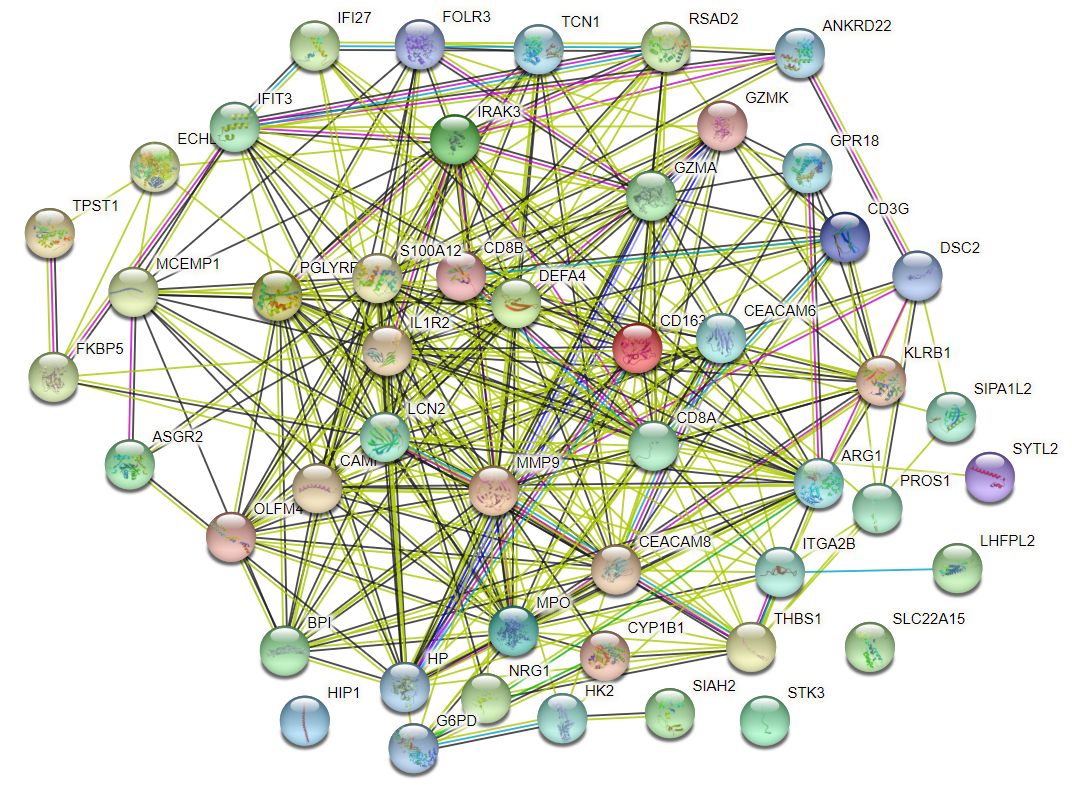


Figure S3. Protein-protein interaction network of the common genes between MDD and IS. MDD, major depressive disorder; IS, ischemic stroke.


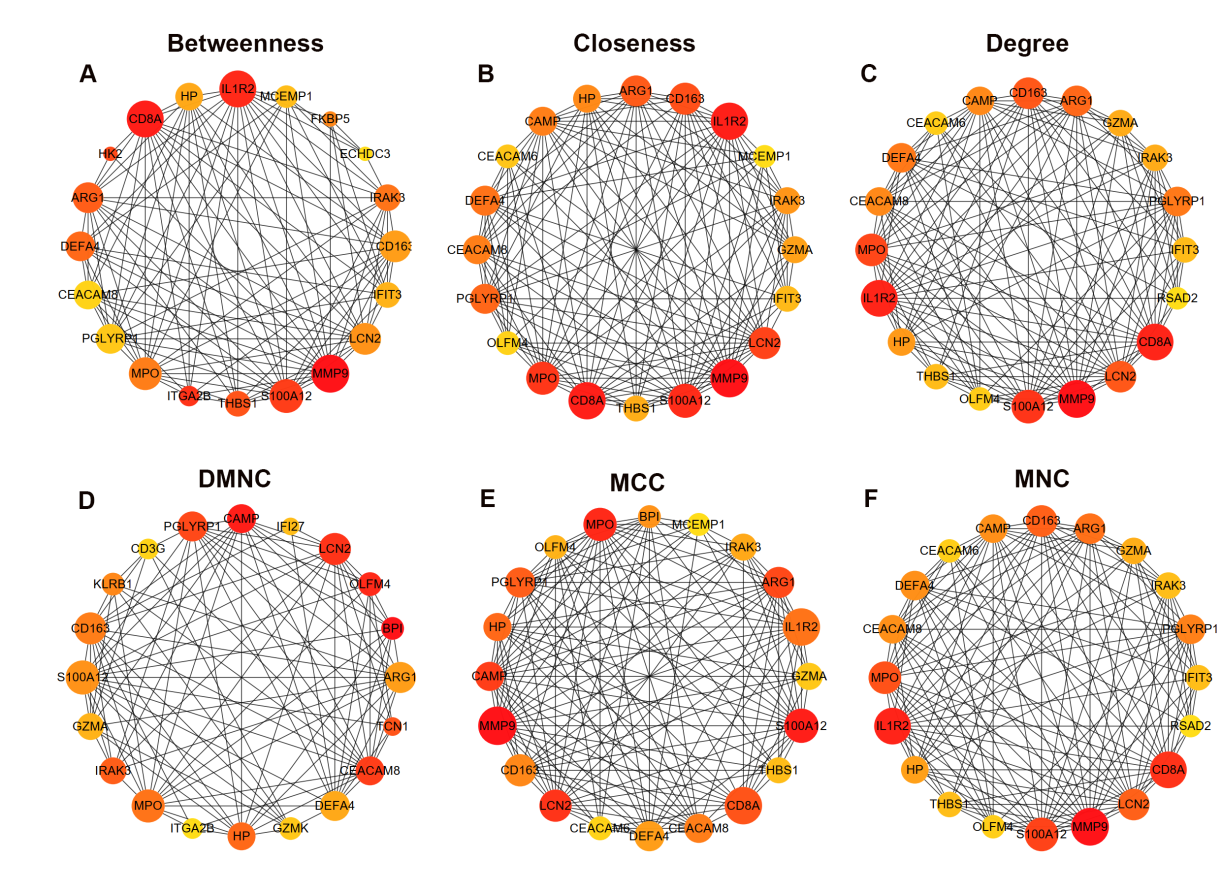


Figure S4. The top 20 genes identified from the PPI network using the Cytohubba plug in Cytosacpe.
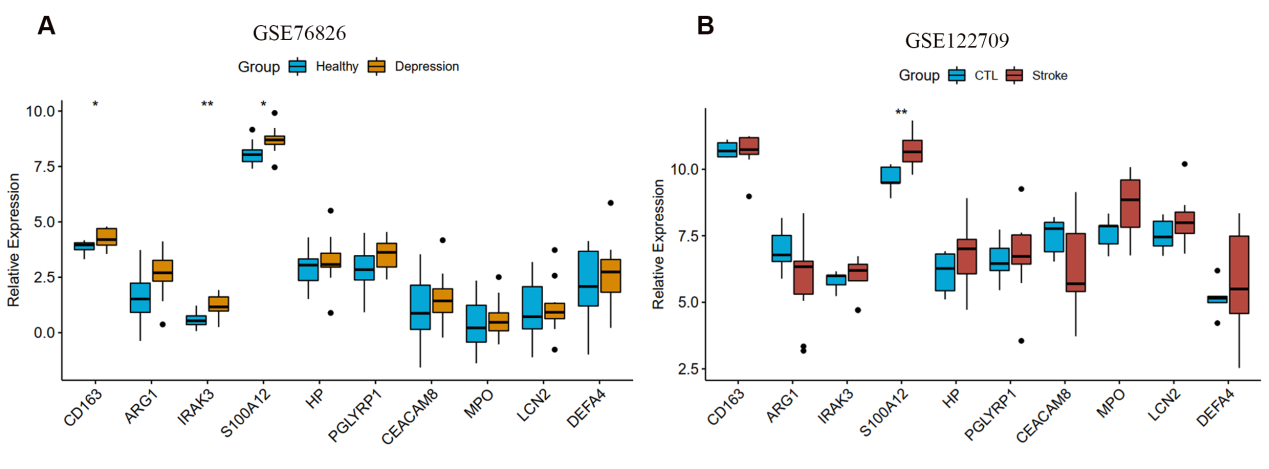


Figure S5. S100A12 was validated as the only differentially expressed gene among ten hub common DEGs between MDD major depressive disorder and IS in other datasets. (A) The expression levels of ten hub genes in MDD in GSE76826 dataset. (B) The expression levels of ten hub genes in IS in GSE122709 dataset. MDD, major depressive disorder; IS, ischemic stroke. *p‐value < .05, **p‐value < .01.

Table S1. The detail information and descriptions of other 39 genes.

| Gene name | Ensembl ID | Gene description | Chromosome | Group |
| --- | --- | --- | --- | --- |
| GPR18 | ENSG00000125245 | G protein-coupled receptor 18 | 13 | DOWN |
| CD8B | ENSG00000172116 | CD8b molecule | 2 | DOWN |
| KLRB1 | ENSG00000111796 | killer cell lectin like receptor B1 | 12 | DOWN |
| GZMK | ENSG00000113088 | granzyme K | 5 | DOWN |
| CD3G | ENSG00000160654 | CD3g molecule | 11 | DOWN |
| CD8A | ENSG00000153563 | CD8a molecule | 2 | DOWN |
| SYTL2 | ENSG00000137501 | synaptotagmin like 2 | 11 | DOWN |
| GZMA | ENSG00000145649 | granzyme A | 5 | DOWN |
| STK3 | ENSG00000104375 | serine/threonine kinase 3 | 8 | UP |
| LHFPL2 | ENSG00000145685 | LHFPL tetraspan subfamily member 2 | 5 | UP |
| SLC22A15 | ENSG00000163393 | solute carrier family 22 member 15 | 1 | UP |
| CYP1B1 | ENSG00000138061 | cytochrome P450 family 1 subfamily B member 1 | 2 | UP |
| G6PD | ENSG00000160211 | glucose-6-phosphate dehydrogenase | X | UP |
| MMP9 | ENSG00000100985 | matrix metallopeptidase 9 | 20 | UP |
| HIP1 | ENSG00000127946 | huntingtin interacting protein 1 | 7 | UP |
| DSC2 | ENSG00000134755 | desmocollin 2 | 18 | UP |
| HK2 | ENSG00000159399 | hexokinase 2 | 2 | UP |
| PROS1 | ENSG00000184500 | protein S | 3 | UP |
| ANKRD22 | ENSG00000152766 | ankyrin repeat domain 22 | 10 | UP |
| THBS1 | ENSG00000137801 | thrombospondin 1 | 15 | UP |
| ECHDC3 | ENSG00000134463 | enoyl-CoA hydratase domain containing 3 | 10 | UP |
| TPST1 | ENSG00000169902 | tyrosylprotein sulfotransferase 1 | 7 | UP |
| FOLR3 | ENSG00000110203 | folate receptor gamma | 11 | UP |
| MCEMP1 | ENSG00000183019 | mast cell expressed membrane protein 1 | 19 | UP |
| IL1R2 | ENSG00000115590 | interleukin 1 receptor type 2 | 2 | UP |
| SIPA1L2 | ENSG00000116991 | signal induced proliferation associated 1 like 2 | 1 | UP |
| TCN1 | ENSG00000134827 | transcobalamin 1 | 11 | UP |
| FKBP5 | ENSG00000096060 | FKBP prolyl isomerase 5 | 6 | UP |
| ASGR2 | ENSG00000161944 | asialoglycoprotein receptor 2 | 17 | UP |
| ITGA2B | ENSG00000005961 | integrin subunit alpha 2b | 17 | UP |
| OLFM4 | ENSG00000102837 | olfactomedin 4 | 13 | UP |
| NRG1 | ENSG00000157168 | neuregulin 1 | 8 | UP |
| CEACAM6 | ENSG00000086548 | CEA cell adhesion molecule 6 | 19 | UP |
| SIAH2 | ENSG00000181788 | siah E3 ubiquitin protein ligase 2 | 3 | UP |
| BPI | ENSG00000101425 | bactericidal permeability increasing protein | 20 | UP |
| RSAD2 | ENSG00000134321 | radical S-adenosyl methionine domain containing 2 | 2 | UP |
| IFIT3 | ENSG00000119917 | interferon induced protein with tetratricopeptide repeats 3 | 10 | UP |
| IFI27 | ENSG00000275214 | interferon alpha inducible protein 27 | 14 | UP |
| CAMP | ENSG00000164047 | cathelicidin antimicrobial peptide | 3 | UP |

Table S2. Summary of pathway and process enrichment analysis.

| Terms | Category | Description | Count | % | Log10(P) | Log10(q) |
| --- | --- | --- | --- | --- | --- | --- |
| R-HSA-6798695 | Reactome Gene Sets | Neutrophil degranulation | 8 | 80 | -12.78 | -8.43 |
| GO:0031640 | GO Biological Processes | killing of cells of other organism | 3 | 30 | -6.66 | -2.92 |
| GO:0032496 | GO Biological Processes | response to lipopolysaccharide | 4 | 40 | -5.62 | -2.23 |
| GO:0002698 | GO Biological Processes | negative regulation of immune effector process | 3 | 30 | -5.23 | -2.03 |
| R-HSA-449147 | Reactome Gene Sets | Signaling by Interleukins | 3 | 30 | -3.4 | -0.47 |
| GO:0006954 | GO Biological Processes | inflammatory response | 3 | 30 | -3.29 | -0.38 |

Table S3. Summary of enrichment analysis in DisGeNET

| Terms | Description | Count | % | Log10(P) | Log10(q) |
| --- | --- | --- | --- | --- | --- |
| C0235574 | Intravascular hemolysis | 4 | 40 | -9.5 | -5.4 |
| C0376618 | Endotoxemia | 6 | 60 | -9 | -5.1 |
| C4082937 | Necrotizing enterocolitis in fetus OR newborn | 5 | 50 | -8.4 | -4.6 |
| C1536220 | ST segment elevation myocardial infarction | 5 | 50 | -7.9 | -4.2 |
| C0004623 | Bacterial Infections | 6 | 60 | -7.9 | -4.2 |
| C0014038 | Encephalitis | 5 | 50 | -7.5 | -3.9 |
| C0742343 | Acute Chest Syndrome | 5 | 50 | -7 | -3.4 |
| C1848296 | DOSAGE-SENSITIVE SEX REVERSAL | 4 | 40 | -6.8 | -3.3 |
| C0021368 | Inflammation | 5 | 50 | -6.7 | -3.2 |
| C0011195 | Dejerine-Sottas Disease (disorder) | 4 | 40 | -6.6 | -3.2 |
| C0240066 | Iron deficiency | 4 | 40 | -6.6 | -3.2 |
| C2609414 | Acute kidney injury | 4 | 40 | -6.6 | -3.2 |
| C0038525 | Subarachnoid Hemorrhage | 5 | 50 | -6.5 | -3.2 |
| C0035204 | Respiration Disorders | 4 | 40 | -6.4 | -3.1 |
| C0577631 | Carotid Atherosclerosis | 4 | 40 | -6.3 | -3 |
| C0042164 | Uveitis | 4 | 40 | -6.1 | -2.8 |
| C3714636 | Pneumonitis | 5 | 50 | -5.8 | -2.6 |
| C0856169 | Endothelial dysfunction | 5 | 50 | -5.8 | -2.6 |
| C2347126 | Microscopic Polyarteritis | 3 | 30 | -5.7 | -2.6 |
| C0035126 | Reperfusion Injury | 4 | 40 | -5.7 | -2.6 |

Table S4. Prediction of drugs interacted with hub genes in DGidb database.

| Gene name | Drug | Interaction types | Sources | PMIDs |
| --- | --- | --- | --- | --- |
| ARG1 | CHEMBL1099168 | | DTC | 20441173 |
| ARG1 | CHEMBL1099167 | | DTC | 20441173 |
| S100A12 | ATOGEPANT | | TTD |  |
| S100A12 | RIMEGEPANT | | TTD |  |
| S100A12 | METHOTREXATE | | NCI | 15077313 |
| S100A12 | EPTINEZUMAB | | TTD |  |
| S100A12 | UBROGEPANT | | TTD |  |
| HP | STREPTOZOCIN | | NCI | 16713232 |
| HP | PYRIDOXINE | | NCI | 6935482 |
| MPO | VERDIPERSTAT | inhibitor | GuideToPharmacology | |
| MPO | PF-06282999 | inhibitor | GuideToPharmacology | |
| MPO | DIMETHYL SULFOXIDE | | NCI | 1845843 |
| MPO | PSORALEN |  | NCI | 15865234 |
| MPO | TOLMETIN |  | NCI | 6266970 |
| MPO | DICLOFENAC | | NCI | 2173589 |
| MPO | DOXYCYCLINE | | NCI | 14564835 |
| MPO | ASULACRINE | | NCI | 1333205 |
| MPO | NIMESULIDE | | NCI | 17176264 |
| MPO | PYRAZINAMIDE | | NCI | 2832129 |
| MPO | PROPYLTHIOURACIL | | DTC | 26509551 |
| MPO | FLUDARABINE | | NCI | 15608444 |
| MPO | LORATADINE | | NCI | 17159802 |
| MPO | OCTREOTIDE | | NCI | 15003363 |
| MPO | TRIMETHOPRIM | | NCI | 7425598 |
| MPO | THEOPHYLLINE | | NCI | 8630596 |
| MPO | LITHIUM |  | NCI | 8224362 |
| MPO | LIDOCAINE | | NCI | 8973808 |
| MPO | TENECTEPLASE | | NCI | 16650886 |
| MPO | FLUTAMIDE | | NCI | 16330533 |
| MPO | FENTANYL |  | NCI | 8391745 |
